# MiR-595 and Cldnd1: Potential related factors for bone loss in postmenopausal women with hip osteoporotic fracture

**DOI:** 10.1371/journal.pone.0313106

**Published:** 2024-12-31

**Authors:** Sun Jingyue, Liu Peixin, Wang Xiao

**Affiliations:** 1 Department of Oncology, The First Affiliated Hospital of Soochow University, Suzhou, Jiangsu, China; 2 Department of Orthopaedics, Suzhou Xiangcheng People’s Hospital, Suzhou, Jiangsu, China; 3 Department of Orthopaedics, The Second Affiliated Hospital of Soochow University, Suzhou, Jiangsu, China; Huashan Hospital Fudan University, CHINA

## Abstract

**Background:**

Recently researches have reported that miRNA and its target genes are associated with osteoporosis. MiRNA and mRNA might be potential diagnostic markers for osteoporosis.

**Purposes:**

The aim of this study is to explore the potential miRNA and mRNA markers by bioinformatics method and clinical analysis.

**Patients and methods:**

The miRNA expression profiles were obtained from GSE74209, GSE64433 and GSE115773 in Gene expression Omnibus (GEO). The mRNA expression profiles were obtained from GSE100609. Wayne intersection were used to explore the different expressed miRNAs (DE-miRs). Select the miRNA with the highest Fold Change for subsequent research. Screening of miRNA target genes using TargetScan and miRDB tools. GO and KEGG analyses of target genes (TGs) function were performed. Validate the selected TGs in the GSE100609. We collected female patients with femural intertrochanteric fractures from July 1, 2023 to October 31, 2023. Patient’s bone marrow and clinical data were collected. MiRNA and the target mRNA differentially expressed in bone marrow were verified through RT-qPCR. All data were subjected to Shapiro-Wilk test. Using Pearson or Spearman test to detect the correlation between various indicators, and then incorporating indicators related to bone density into multiple linear regression equations. Partial correlation analysis was used to analyze the correlation between the final indicators and bone density.

**Results:**

A total of 140 DE-miRs were identified. Set the fold change to “>1” and ultimately include 5 miRNAs. Using miR-595 (highest |log_2_ FC|) as the subject of subsequent research. 3542 targeted mRNAs were predicted from TargetScan and 362 were from miRDB. 337 TGs were intersected, which were mainly enriched in nucleus. Only Cldnd1 were identified using the GSE100609 dataset. We found that miR-595 was highly expressed in patients with high bone mass, while Cldnd1 was downregulated. There was a strong collinearity between miR-595 and Cldnd1. Further multiple linear regression analysis showed a high correlation between miR-595 and bone density.

**Conclusions:**

These data suggest that miR-595 and Cldnd1 might be related factors for decreased bone mass.

## Introduction

Osteoporosis is a systemic disease characterized by bone metabolism disorders, unstable bone resorption and formation. Its characteristic is a decrease in bone quality, a decrease in bone trabeculae, and the occurrence of brittle fractures [1]. The incidence rate is higher in the elderly, especially in postmenopausal women [2]. The elderly population is prone to falls and then to brittle fractures due to certain reasons such as decreased vision and hearing, weakened muscle strength, and delayed movement [3]. Osteoporosis fracture (also known as brittle fracture) refers to a fracture that occurs after minor trauma (equivalent to falling from a standing height or lower), and is the most serious consequence of osteoporosis [4]. The common locations of osteoporotic fractures include the vertebral body, distal forearm, hip, proximal humerus, and pelvis, among which vertebral and hip fractures are the most common [5].

Many studies have shown that osteoporosis is associated with microRNA (miRNA). miRNA is an endogenous single stranded, non coding small RNA [6]. By binding to the 3’ untranslated region (UTR) of the target mRNA, it participates in regulating approximately 30% of gene expression and is an important post transcriptional level regulatory factor [7]. miRNA plays a wide range of roles in physiological processes such as cell proliferation, differentiation, apoptosis, and metabolism [8]. They are critical post-transcriptional regulators of gene expression that control osteoblast mediated bone formation (eg, miR-218) and osteoclast-related (eg, miR-148a) bone remodeling [9]. Each miRNA stimulates differentiation by suppressing inhibitory signaling pathways.

Plasma miRNAs were suggested as sensitive biomarkers for postmenopausal osteoporosis (PMO). Li et al have reported a correlation between miR-21, miR-133a and bone mineral density (BMD) [10]. miRNAs extracted from femoral neck trabecular bone from women undergoing hip replacement due to either osteoporotic fracture or osteoarthritis in the absence of osteoporosis were analyzed by Laura et al, and finally miR-320a and miR-483-5p were confirmed [11]. However, these studies only explored the correlation between miRNA and osteoporosis, and did not investigate whether the downstream of miRNAs, or called target genes (TGs) are also involved. Therefore, there are certain limitations to these researches.

In the present study, bioinformatics methods were used to investigate the differential miRNA expression profiles of PMO samples in the Gene Expression Omnibus (GEO, https://www.ncbi.nlm.nih.gov/geo/)) database, and explore the role of its TGs in PMO. In addition, we explored the potential relationship between miRNA, TGs and BMD. Further attempts were made to identify miRNA and TG as related factors for bone loss among femural intertrochanteric fractures patients, which might be helpful for the diagnosis and treatment of PMO.

## Methods

### Data collection

**Fig 1:**
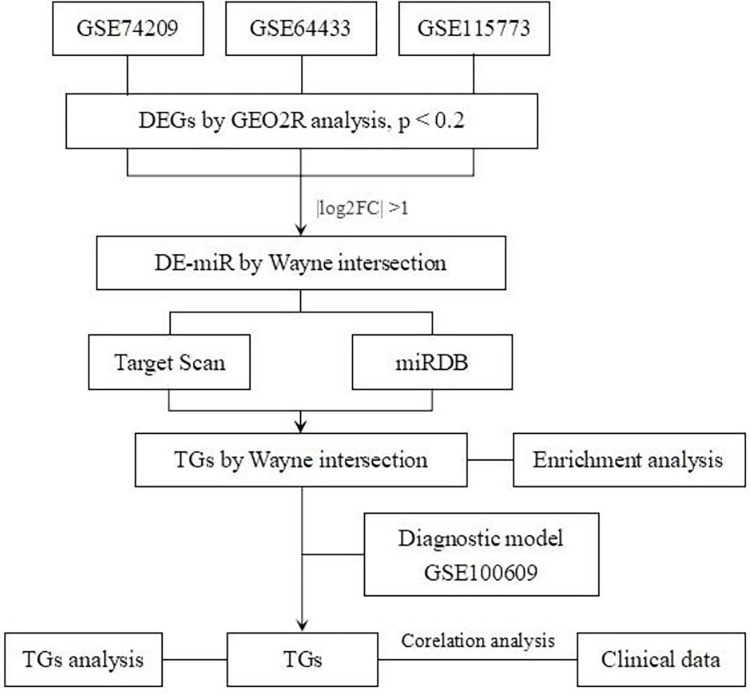
A flowchart showing the research methods used in this study.

The relevant PMO datasets, GSE74209, GSE64433, GSE115773 and GSE100609, were downloaded from the GEO database (detailed information in S1–S4 Files). GSE74209, consists of miRNAs data corresponding to Homo sapiens, with 6 femoral neck trabecular bone obtained from postmenopausal women undergoing hip replacement due to osteoporotic fracture, and 6 form osteoarthritis in the absence of PMO. Both GSE64433 and GSE115773 contain serum miRNAs data collected from postmenopausal women with PMO (low bone mass, LB) or not (high bone mass, HB). While GSE100609 contains differential mRNA between osteoporosis and non-osteoporosis women. The research methods used in this study were listed in Fig 1.

### Differential expression analysis

Use GEO2R (https://www.ncbi.nlm.nih.gov/geo/geo2r/) to compare two groups of samples in order to identify miRNAs or mRNAs that are differentially expressed across experimental conditions [12]. Results are presented as a table of genes ordered by significance, with the threshold of P < 0.2. Different expressed genes (DEGs) with |log_2_ fold change (FC)| > 1 were selected. Then perform Wayne intersection on miRNAs differentially expressed in these three databases (GSE74209, GSE64433, GSE115773) to identify the different expressed miRNAs (DE-miR).

### Target genes analysis

Draw a volcano plot and cluster map of the miRNAs obtained after Wayne intersection, and arrange the differential miRNAs between HB and LB groups in descending order of |log_2_ FC|. Take the miRNA with the highest |log_2_ FC| value for further research. Use the TargetScan database (https://www.targetscan.org/vert_80/) and miRDB database (https://mirdb.org/mirdb/index.html) to predict miRNA target genes as previously reported [13], and perform Wayne intersection again on the mRNA predicted by the two databases.

### GO and KEGG pathway enrichment analyses

GO and KEGG pathway enrichment analyses were conducted to detect the GO terms that displayed enrichment in 3 distinct categories (cellular components, molecular functions, and biological processes) and the KEGG pathways. Incorporate the mRNA screened in the previous step into the DAVID database (https://david.ncifcrf.gov/) for GO and KEGG analysis [14]. The enrichment results were visualized by using enrichment dot bubble. The target mRNAs predicted in the previous step were validated using GSE100609 database to obtain the final TGs.

### Patients with osteoporotic hip fractures

We obtained samples from 10 postmenopausal women patients with intertrochanteric fracture who underwent internal fixation (proximal femoral nail antirotation, PFNA), between July 1, 2023 and October 31, 2023. Evans’ classification was type I or II. We divided 10 patients into high bone mass group (HB) and low bone mass group (LB) based on the median of BMD. General data, coagulative function, liver & renal function, blood routine, inflammatory markers, electrolytes, anemia markers and bone metabolic markers were collected.

### Collection of bone marrow specimens

When the patient underwent PFNA surgery, we inserted a guide needle through the opening at the apex of the greater trochanter of the femur, and then extended a negative pressure suction tube along the guide needle into the proximal femoral medullary cavity to extract 5ml of bone marrow tissue. Immediately extract RNA from bone marrow tissue for subsequent research. This study follows the GCP principle and follows the protocol approved by the ethics committee to conduct clinical research, protecting the health and rights of subjects. The relevant operations of this study have been approved by all subjects and signed an informed consent form. This study has been approved by the ethics committee of The Second Affiliated Hospital of Soochow University (No. JD-LK-2022-061-01).

### Real-time fluorescent quantitative polymerase chain reaction (PCR) analysis

Total RNA was extracted from bone marrow tissues from these patients, and RNA was reverse transcribed into cDNA using a reverse transcription kit (Invitrogen) following the manufacturer’s instructions. Real-time PCR was performed with an Applied Biosystems 7900HT system (Applied Biosystems) using a SYBR Premix Ex TaqTM kit (Takara). Each experiment was performed in duplicate, and the results were standardized. Data were expressed as the fold-change relative to the control. Primer sequences were designed with Primer Premier software (Premier Biosoft). The primers used in this study were:

hsa-miR-595, 5′-ACGGTCCCTTGGAGTTTTC-3′ (FW) and 5′-AATGGCTCACCCAAACTGC-3′ (RV); Cldnd1, 5′-CTAACTGAGCAGTTCATGGAG-3′ (FW) and 5′-TAAGCTTCGGCAAATGCAAG-3′ (RV).

### Statistical analysis

All the quantitative data were presented as the mean ± standard deviation (SD). Using Shapiro-Wilk test to detect whether each indicator was normally distributed. We analyzed the correlation between normal distribution data and BMD using Pearson test. While the correlation between non-normal distribution data and BMD was analyzed using Spearman test. Then we screened out indicators significantly related to BMD. The variance inflation factor (VIF) was used to represent the collinearity of these indicators, which were further analyzed via Spearman correlation test. After removing collinearity indicators, BMD was taken as the dependent variable, and the remaining indicators were included in the multiple linear regression equation. After deleting indicators with P > 0.05, the remaining indicators were included in multiple linear regression analysis again. Finally, partial correlation analysis was used to detect the correlation between miRNA and BMD. GraphPad Prism software and bioinformatics (http://www.bioinformatics.com.cn/) were used for artwork creating. SPSS 21.0 statistical software (IBM Corp) was used for data analysis. All tests were two-sided, and P values < 0.05 were considered statistically significant.

## Results

### Expression of DE-miRs in PMO

**Fig 2:**
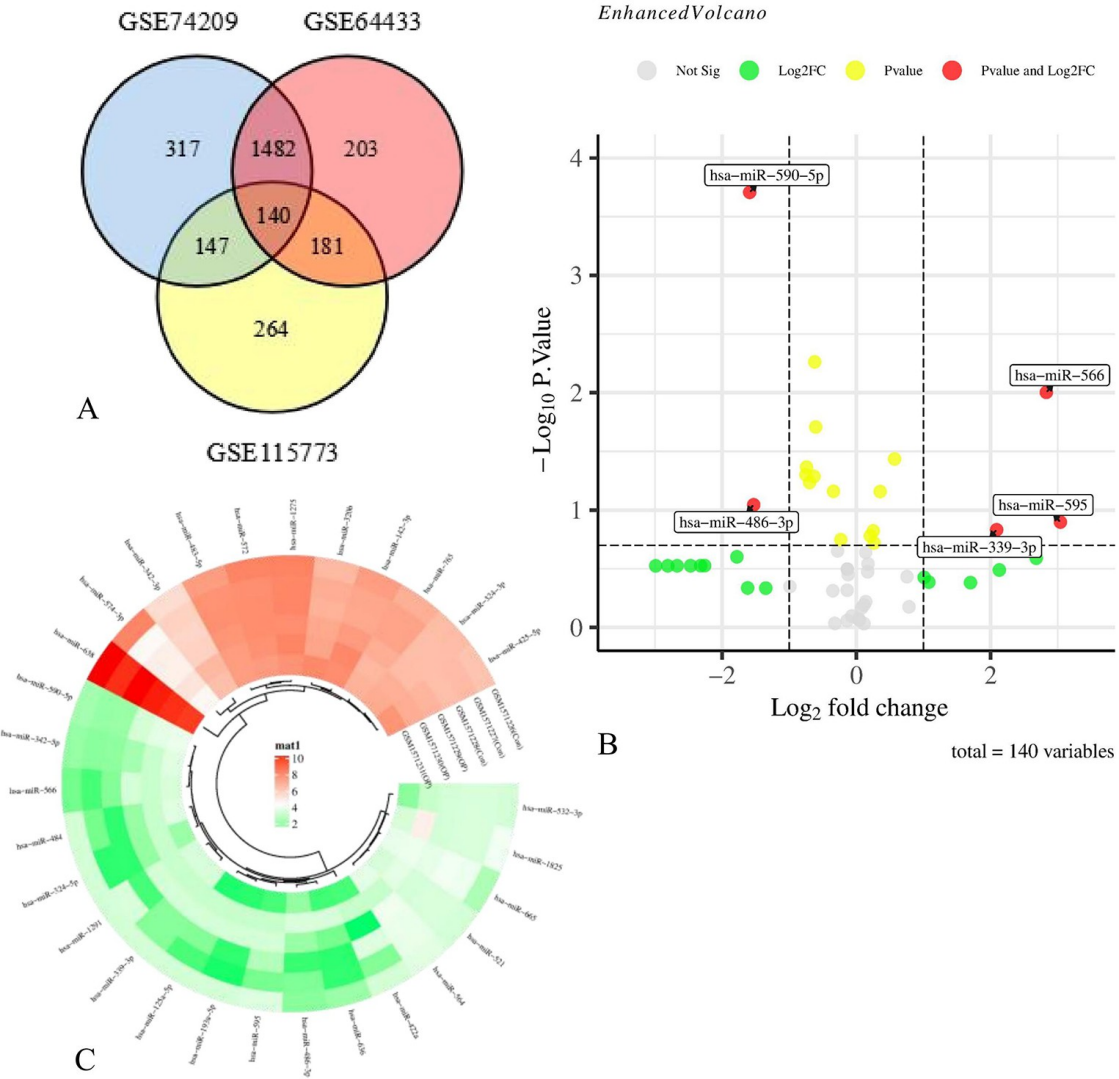
Differentially expression analysis of DE-miRs. A. Wayne intersection of three databases; B. A volcano plot of the 140 different expressed miRNAs; C. A heat map of the DE-miRNAs identified in the GSE64433 dataset.

Fig 1 showed the flowchart associated with the analysis of DE-miRs expression in PMO. We took the intersection of 2086 miRNAs from GSE74209, 2006 miRNAs obtained from GSE64433 and 732 from GSE115773. 140 miRNAs expressed in all three databases (Fig 2A). Group according to the samples in GSE64433. The differences in miRNAs expression between HB and LB were presented as a volcano plot in Fig 2B, and screened them with |log_2_ FC| > 1, then the results were presented in a heatmap in Fig 2C. Based on the results of the volcano plot, we arranged miRNAs in order of |log_2_ FC| values from high to low, and selected hsa-miR-595 with the highest |log_2_ FC| value as the subsequent research object.

### Prediction of target mRNAs and enrichment analyses

**Fig 3:**
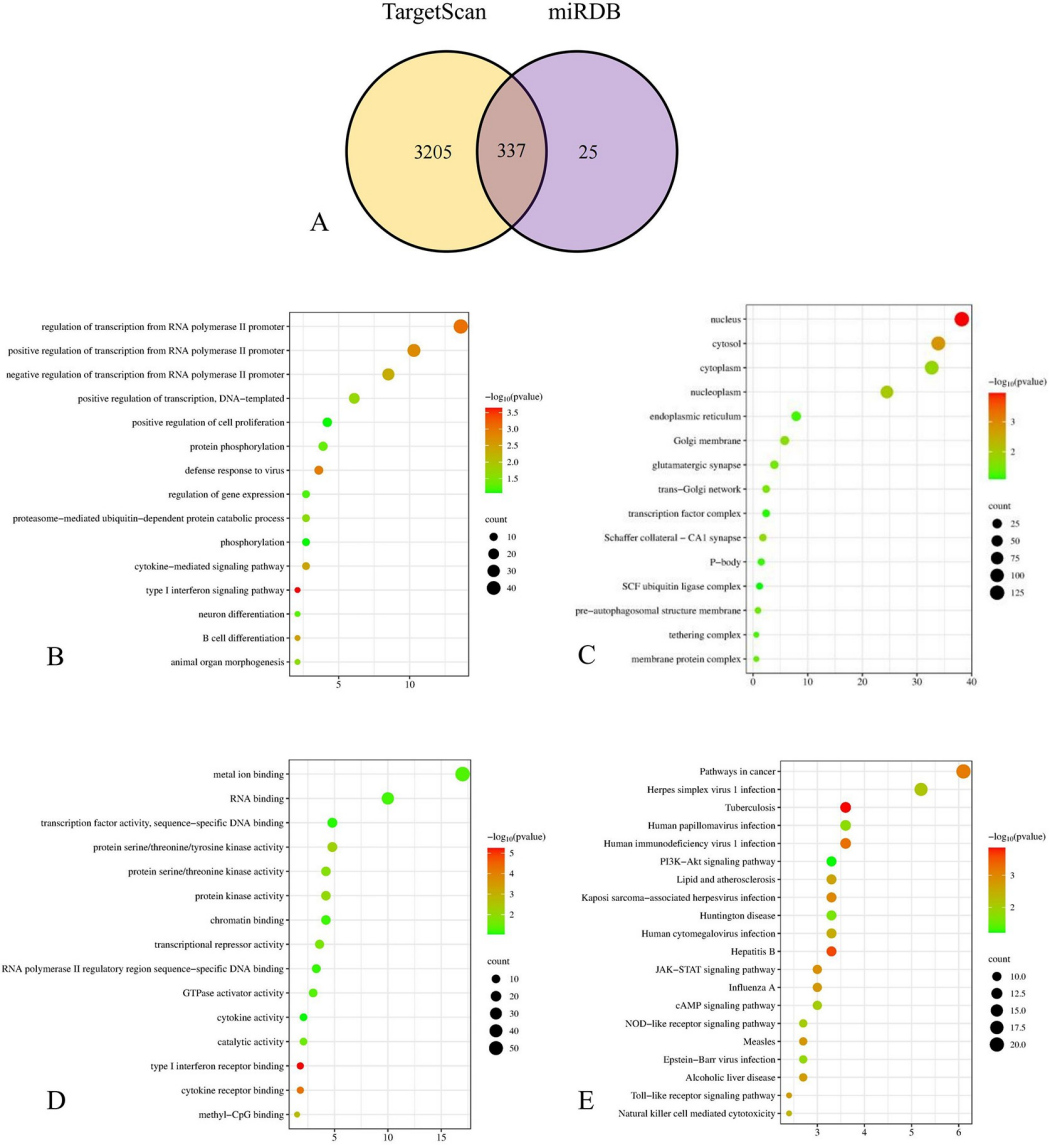
Functional enrichment analysis of predicted target genes. A. Wayne intersection of TGs predicted from TargetScan and miRDB; B. BP annotation of TGs. C. CC annotation of TGs. D. MF annotation of TGs. E. KEGG annotation of TGs.

3542 mRNA predictions were made using TargetScan and 362 predictions were made via miRDB. We screened 337 mRNAs from them (Fig 3A). To further explore the enrichment pathways and functions of these mRNAs, we entered the mRNAs list into DAVID tool and obtained data for GO analysis and KEGG pathway analysis. These genetic biological processes (BP) were mainly concentrated in the regulation of transcription from RNA polymerase II promoter, especially in the positive one. The cellular components (CC) of the mRNAs were mostly localized to the nucleus and cytosol. The molecular function (MF) were mainly related to protein binding and metal ion binding (Fig 3B–3D). Besides the pathways in cancer, the KEGG pathway analysis also enriched in Herpes simplex virus 1 infection and tuberculosis (Fig 3E).

**Fig 4:**
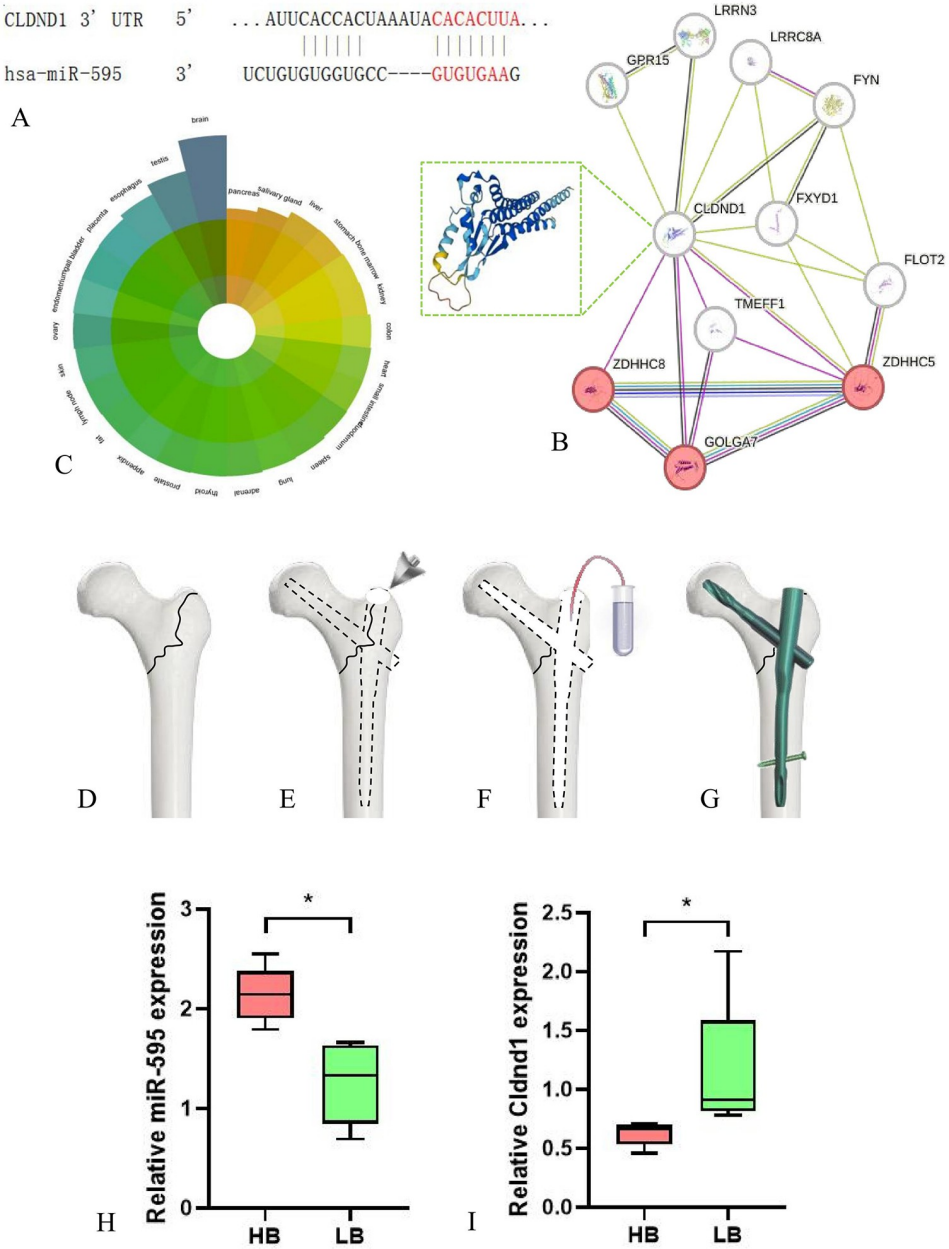
Verification of miRNA and target gene. A. The combination of hsa-miR-595 and Cldnd1 3’UTR; B. PPI network of Cldnd1; C. The distribution of Cldnd1 in various organs of the human body; D-G. Flow chart of femoral bone marrow extraction for patients undergoing PFNA surgery. After the opening of the apex of the greater trochanter of the femur, a negative pressure suction tube is inserted into the medullary cavity to extract bone marrow; H. The expression of miR-595 in the HB and LB group; I. The expression of Cldnd1 in two groups.

We conducted preliminary validation on 337 predicted mRNAs using the GSE100609 database. Interestingly, out of the 337 predicted genes, only claudin domain containing 1 (Cldnd1) appeared in the database of GSE100609. The combination of hsa-miR-595 and Cldnd1 3’UTR was shown in Fig 4A. Protein−protein interaction (PPI) network of Cldnd1 was conducted through the STRING database (https://cn.string-db.org/), and the results showed that Cldnd1 was enriched in protein palmitoylation (GO:0018345) (Fig 4B). Cldnd1 can be expressed in multiple organs in the human body, and Gene database (https://www.ncbi.nlm.nih.gov/gene/56650) reports that this gene can be expressed in bone marrow (Fig 4C). Therefore, we collected female patients with femural intertrochanteric fractures and extracted their bone marrow during surgery (Fig 4D–4G). Divided 10 patients into 2 groups based on the hip T-value detected by DXA. RT-qPCR was used to detect the expression levels of miR-595 and Cldnd1 in two groups of patients for final validation. Patients with lower bone mass showed lower expression of miR-595 and higher expression of Cldnd1, with statistically significant differences (Fig 4H, 4I).

### Clinical data

S1 Table in S5 File showed the clinical data of PMO patients in HB and LB groups. General data, coagulative function, liver & renal function, blood routine, inflammatory markers, electrolytes, anemia markers and bone metabolic markers were listed. The fold-increases of indicators of HB group were 1.24- (weight), 3.20- (FIB), 1.85- (ALT), 1.51- (γ-GGT), 1.41- (ChE), 2.35- (EosR), 1.53- (HCT), 1.36- (GFR), 1.41- (TC), 1.98- (VitB12), 1.51- (EPO), 1.56- (1,25-OH2D) fold, respectively. While other indicators further decreased to 1.50- (TBiL), 2.40- (DBiL), 1.38- (CK), 1.32- (LDH), 1.45- (Lym), 3.55- (CRP), 1.48- (ER), 1.57- (Cre), 2.99- (Fer), 1.50- (CT), 2.53- (P1NP), 1.60- (β-CTx) fold, respectively, compared to LB group.

### Correlation analysis

**Fig 5:**
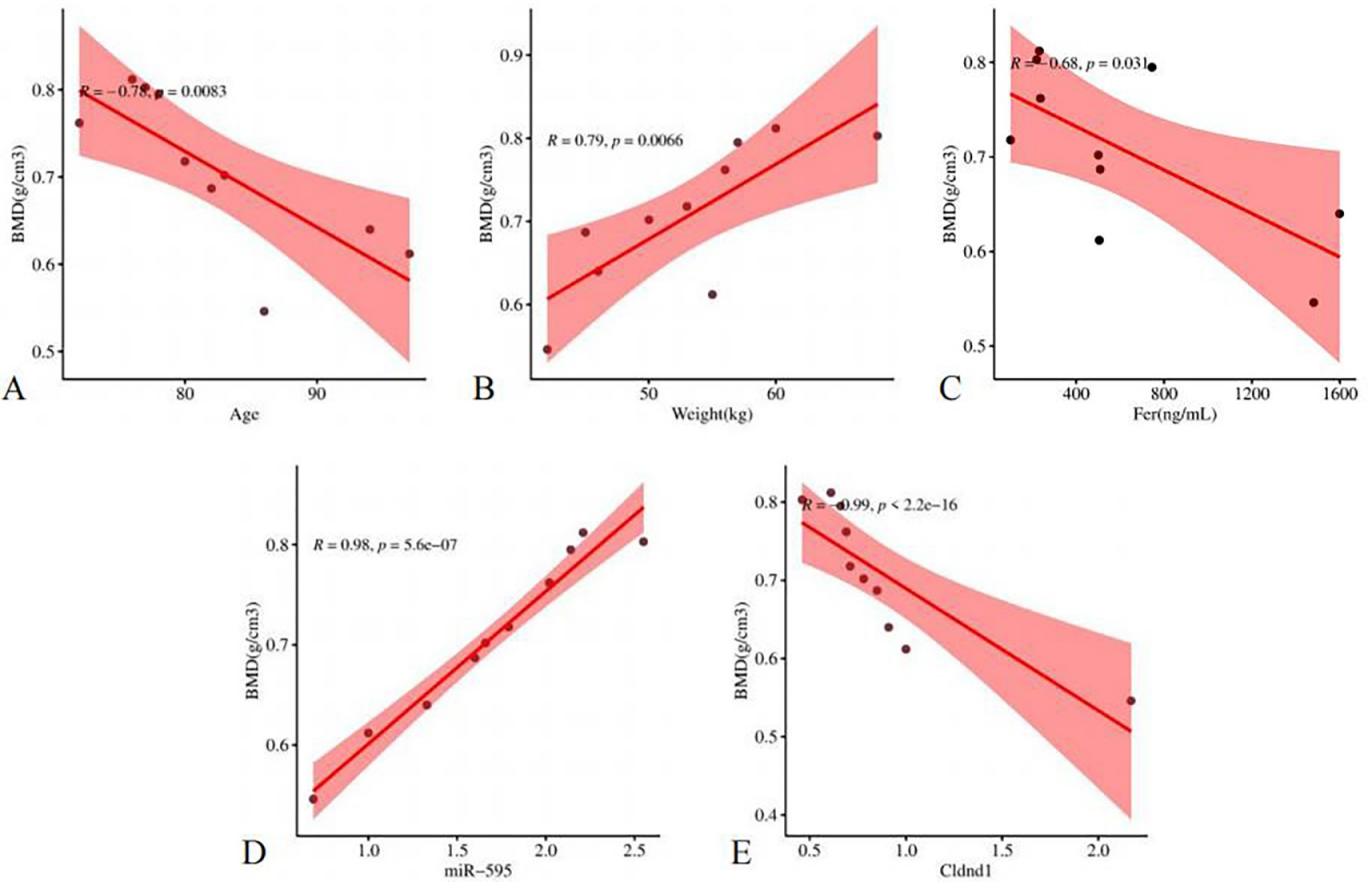
Scatter plot of some highly correlated indicators, including Age (A), Weight (B), Fer (C), miR-595 (D), Cldnd1 (E).

All quantitative data were tested for normal distribution using the Shapiro-Wilk test. The results indicated that TT, FIB, TBiL, DBiL, γGGT, PDW, P1NP, Cldnd1, β-CTx, CT, Eos, MCV, VitB12, IBiL, Fer, AT, ALP, CRP, AG, Mono, Bas, EPO, IFNA4, APTT were non-normal distribution. Other indicators are normally distributed. To further analyze the univariate correlation variables with BMD, we used Pearson correlation analysis for normal distribution data and Spearman correlation analysis for non-normal distribution data (Tables 1 and 2). The scatter plot of the correlation between the selected indicators (Age, Weight, miR-595, Cldnd1, Fer) and BMD was shown in Fig 5. According to the scatter plot results, all indicators showed a linear trend with BMD. To further test the correlation, we used multiple linear regression equations. Before conducting linear regression analysis, we evaluated the collinearity between five indicators. Unfortunately, these independent variables were collinear with each other (S2 Table in S5 File). In this study, the correlation coefficient between miR-595 and Cldnd1 was -0.873 (p = 0.003). Therefore, we excluded Cldnd1 and analyzed whether there was collinearity between the remaining 4 indicators. After removing Cldnd1, there was no collinearity between the indicators, VIFs were all less than 10 (S3 Table in S5 File). The first multiple linear regression equation excluded Weight and Fer (Table 3). We included Age and miR-595 again in the multiple linear regression equation, and the results showed a significant correlation between both and BMD (Table 4). We then controlled for the variable Age and conducted a partial correlation analysis between miR-595 and BMD. The correlation coefficient was 0.953, p<0.001, indicating that miR-595 might be a related factor for BMD decline.

**Table 1:** Pearson correlation analysis for normal distribution data.

	r	p
Age	-0.777	0.008
Weight	0.790	0.007
miR-595	0.981	<0.001

**Table 2:** Spearman correlation analysis for non-normal distribution data.

	r	p
Cldnd1	-0.988	<0.001
Fer	-0.636	0.048

**Table 3:** Multiple linear regression.

	r	p
Age	3.032	0.023
Weight	0.010	0.992
miR-595	4.432	0.004
Fer	-0.162	0.877
R^2^ = 0.998		

**Table 4:** Multiple linear regression.

	r	p
Age	13.310	<0.001
miR-595	13.536	<0.001
R^2^ = 0.998		

## Discussion

Traditional views suggest that age, weight, hormone levels, dietary habits and exercise are related to bone metabolism [15]. However, recent studies suggest that intestinal microbiota imbalance, inflammation, iron accumulation and ferroptosis, and inhibition of angiogenesis are also involved in the occurrence and development of osteoporosis [16]. Therefore, osteoporosis is not a simple disease with abnormal bone metabolism, but a systemic disease. Exploring new causes of osteoporosis has important clinical significance for the prevention and treatment of osteoporosis. Especially the latest guideline has included hip and vertebral fragility fractures as diagnostic criteria for osteoporosis, without the need to test their T-values, resulting in a significant increase in the number of osteoporosis patients worldwide [17]. Therefore, early screening for PMO is particularly important.

MiRNAs are small noncoding RNA molecules of about 20 nucleotides implicated in posttranscriptional gene expression regulation through targeting of mRNAs [18]. A strand of miRNA will bebound by Argonaut 2 protein that direct the binding of miRNA to 3’UTR of mRNA, determining inhibition of expression of gene target. In brief, This axis composed of miRNA and mRNA binding inhibits TGs translation or enhances their degradation. Ji et al analyzed to get Palbociclib/miR-141-3p/STAT4 axis through miRNA-mRNA network and drug sensitivity analysis, which can be used for the prevention and treatment of osteoporosis [19]. Ding et al found that miR-224-5p negatively regulated osteogenic differentiation by targeting Runx2 and Sp7. It also highlights the potential use of miR-224-5p as a therapeutic target and diagnostic biomarker for osteoporosis [20]. In this study, we also utilized existing databases for bioinformatics analysis to explore the early warning effect of miRNA/mRNA axis on bone mass decline.

We searched the GEO database for relevant data on miRNA and osteoporosis, and ultimately included three databases. The specimen sources of these three ones include both bone tissue and whole blood. We believe that if miRNAs are detected only in peripheral blood but not expressed in bone tissue, they cannot directly affect bone metabolism. Therefore, after screening, the final intersection of miRNAs can be detected in both peripheral blood and bone tissue, which better reflects their impact on bone metabolism and facilitates the extraction of peripheral blood as a diagnostic indicator. Due to the limited sample size, setting the P value to 0.05 might miss some genes that were actually differentially expressed. Thus, considering that there might be omissions in the miRNAs after the intersection of three databases, we relaxed the boundary of P value and set it to 0.2 [1]. Race has an impact on BMD. In this study, the samples in GSE74209, GSE115773 and GSE64433 were from Spanish, Mexican, and Chinese women. The sample source of GSE64433 and the subjects in this study were both Chinese women Therefore, GSE64433 was selected as the follow-up research object, and miR-595 with the largest FC value was screened in this database.

As mentioned above, miRNAs need to further generate biological functions by regulating downstream target genes. Therefore, we predicted the downstream target genes of miR-595. We used TargetScan and miRDB tools for prediction and intersection, and finally included 337 mRNAs. After GO analysis of these mRNAs, we found that downstream target genes are mostly enriched in regulation of transcription from RNA polymerase II promoter, protein binding, and metal ion binding. These results suggested that predicted miRNAs were involved in molecular regulation.

To further analyze potential downstream target genes involved in bone metabolism, we validated them again using the GEO database. In the GSE100609 database, out of all predicted 337 mRNAs, only Cldnd1 showed significant differences. By searching for information related to Cldnd1, we found the presence of Cldnd1 in bone marrow tissue [21]. Therefore, we collected 10 elderly female patients with femural intertrochanteric fractures and planned to collect bone marrow during surgery for verification. According to the current diagnostic criteria for osteoporosis, patients with hip fractures caused by low-energy injuries can be diagnosed with osteoporosis [22]. Therefore, when grouping, we divided these 10 osteoporosis patients into two groups based on the hip bone density (T value). According to the PCR results, there were significant differences in miR-595 and Cldnd1 between the HB and LB groups.

After conducting normal distribution tests on the data, we used Pearson correlation analysis and Spearman correlation analysis to detect the correlation between various indicators and BMD. In addition to the miR-595 and Cldnd1, Age, Weight, and Fer were also correlated with BMD. It is widely recognized that age, weight, and bone density are related. Currently, a large number of studies have suggested that iron accumulation could exacerbate the occurrence and development of PMO. Therefore, the results obtained from the single factor correlation analysis in this study are reliable. To explore the related factors of diseases, multiple regression analysis is needed. We plotted scatter plots of these five indicators with BMD, and the results showed that all of these indicators were linearly correlated with BMD. Before conducting multiple linear regression analysis, we tested the collinearity of the five indicators mentioned above. Unfortunately, the results indicated that the VIF was greater than 10. Considering that there might be a correlation between Cldnd1 and miR-595. We temporarily excluded Cldnd1 and retested the remaining four variables for collinearity. The results indicate that the above variables have small collinearity and can be included in the multiple linear regression equation. After two rounds of regression analysis, Weight and Fer were excluded. Age is a recognized risk factor for osteoporosis. In this study, we screened the characteristic variables and finally included two variables: Age and miRNA. By using partial correlation analysis and adjusting for the influence of Age in statistical analysis, we found that miR-595 was also correlated with BMD. Therefore, miR-595 might also be an important factor affecting BMD in addition to age.

## Conclusion

In summary, we demonstrated here that miR-595 and Cldnd1 were presented in human bone marrow, and affected bone mass in this study. We elucidated that miR-595 and Cldnd1 might be related factors for PMO. These findings may probably provide new insights into the development of clinical therapies for the prevention of bone loss-related disorders. The research is limited by the the subjects. We collected bone marrow tissue to detect miR-595 and Cldnd1. The bone marrow contains a multitude of cells, making it difficult to ascertain in which cell miR-595 and Cldnd1 are located. Consequently, conducting the luciferase reporter assay in cells proves challenging. Regrettably, we cannot establish the upstream-downstream relationship between miR-595 and Cldnd1. Additionally, our finding was based only on patients with hip fragility fractures and did not consider osteoporosis patients with vertebral fractures, distal radius fractures, and proximal humeral fractures, et al. Therefore, clinical research will be furthered.

## Supporting information

S1 File Series matrix file of GSE64433. (ZIP)

S2 File Series matrix file of GSE74209. (ZIP)

S3 File Series matrix file of GSE115773. (ZIP)

S4 File Series matrix file of GSE100609. (ZIP)

S5 File S1 Table. Clinical data of PMO patients in HB and LB groups. S2 Table. Collinearity analysis (Age, Weight, miR-595, Cldnd1, Fer). S3 Table. Collinearity analysis (Age, Weight, miR-595, Fer).
(XLSX)

## Abbreviation

GSE: gene expression omnibus series
GO: gene ontology
BP: biological process
CC: cellular component
MF: molecular function
KEGG: kyoto encyclopedia of genes and genomes
DAVID: the database for annotation, visualization and integrated discovery
